# Supplementary material for: Research on influencer marketing strategies based on double-layer network game theory
Source: PLoS One. 2025 Sep 2;20(9):e0326252. doi: 10.1371/journal.pone.0326252 (PMC12404498; doi:10.1371/journal.pone.0326252)
Supplement: Attachment — Submitted filename: Response to Reviewers.docx [file pone.0326252.s004.docx]

Dear editor Tyler Horan.

On behalf of my co-authors, we thank you very much for giving us an opportunity to revise our manuscript， we appreciate editor and reviewers very much for their positive and constructive comments and suggestions on our manuscript entitled “Research on Influencer Marketing Strategies Based on Double-Layer Network Game Theory”.(Submission ID PONE-D-24-46619)

We have studied the Reviewer's comments carefully and have made revision which marked in tracked changes in the revised submission file. We have tried our best to revise our manuscript according to the comments. We would like to submit for your kind consideration.

Thank you and best regards.

Yours sincerely.

Corresponding author.

Name: Zeguo Qiu

E-mail: qiuzg@hrbcu.edu.cn

**List of Responses**

Dear editor and reviewers.

Thank you for your letter and for the Reviewers’ comments concerning our manuscnipt entited “Research on Influencer Marketing Strategies Based on Double-Layer Network Game Theory”. ( Submission ID PONE-D-24-46619).Those comments are all valuable and very helpful for revising and improving our paper, as well as the important guiding significance to our researches. We have studied comments carefully and have made correction which we hope meet with approval. Revised portion are marked in blue in the paper. The main corrections in the paper and the responds to the reviewer's comments are as flowing:

**Responses to the Editor’s comments:**

**1. Please ensure that your manuscript meets PLOS ONE's style requirements, including those for file naming.**

**Responses：**I have modified the manuscript with reference to the template format. The wrong format of some references was modified.

**2.Please state what role the funders took in the study.**

**Responses：**”Sponsor Qiu Zeguo is the first author of the thesis. He formulated the main research direction and content of this study and participated in the revision of the manuscript.” were added in cover letter.

**3. Please confirm at this time whether or not your submission contains all raw data required to replicate the results of your study.**

**Responses：**

(1).The original data of the simulation part are reflected in Tab 4 of the paper.

**Tab.4 System Simulation Parameter Setting Table**

| Parameter | *x* | *y* | *C1* | *C2* | *Q1* | *P* | *ρ* | *τ* |
| --- | --- | --- | --- | --- | --- | --- | --- | --- |
| initial value | 0.2 | 0.2 | 10 | 1000 | 100 | 20 | 1 | 1 |
| Parameter | *λ* | *α* | *γ* | *R* | *L* | *δ* | *K* | *q* |
| initial value | 8% | 0.1 | 10% | 0% | 100 | log^Ni^ | 0.1 | 0.7 |

(2). In the process of overhaul, a part of case verification is added. The original data comes from the public data set on the kaggle website. The website is as follows :

[Cosmetics datasets | Kaggle](https://www.kaggle.com/datasets/kingabzpro/cosmetics-datasets)

https://www.kaggle.com/datasets/kingabzpro/cosmetics-datasets

Additional Editor Comments:

1. The language in several sections is overly technical, which might hinder comprehension for readers outside the field.

In Section 3.1, we have revised the technical descriptions and specialized terminology related to the influencer network construction process to enhance accessibility for non-specialist readers while retaining scientific precision. Key modifications include:

**Before modification：**

Research has found that many real-life network structures exhibit power-law characteristics in their degree distributions. With the development of complex network theory, an increasing number of real networks have been confirmed to possess scale-free properties, including user relationship networks on the internet. Both influencers and fans are users on content creation platforms, so a growing Barabási-Albert (BA) network is used to simulate the attention and follow behaviors between fans and influencers. This paper employs the clustering-variable scale-free network proposed by Holme and Kim, adjusting the selection probability *q* for linking to change the clustering coefficients of nodes and networks, thereby exploring the impact of the influencer’s fan base size on cooperative behaviors within the model.

**after revision：**

Empirical studies reveal that degree distributions in many real-world networks exhibit power-law characteristics[44]. With advances in complex network theory, an increasing number of practical networks—including user relationship networks on the internet —have been identified as scale-free[45]. Given that both influencers and their followers are users on content creation platforms, their followership dynamics can be modeled using the Barabási-Albert (BA) network, which incorporates growth and preferential attachment mechanisms.

This study employs the Growing Scale-Free Networks with Tunable Clustering model proposed by Holme and Kim[46] to simulate user relationship networks on content platforms. By adjusting the selection probability q during the edge-linking phase of network growth, we modulate the clustering coefficients of individual nodes and the entire network. A higher q value corresponds to a larger global clustering coefficient, which structurally manifests as a higher proportion of followers concentrated around nodes with greater degrees (i.e., users with more followers). This parameter enables systematic exploration of how influencer follower scale—driven by network topology—shapes cooperative behaviors in subsequent experiments. To construct the influencer network, we retain the top-degree nodes from the simulated user network and record their follower counts.

2. The literature review is heavily skewed towards industry reports and lacks sufficient academic citations.

In view of the problem of writing industry reports, we have deleted many expressions about industry phenomena in the article. For example, the number of Douyin users in 3.1, some content in 2.2.1 that is not related to the model hypothesis, and so on.

**In addition, the hypothesis H5 is re-stated to streamline the content :**

**Before modification：**

Labor cooperation and commission income are mutually independent. Various platforms have functionalities similar to the “selection plaza,” providing a platform for influencers to choose and sell products. Any product in the selection plaza can be listed in an influencer’s showcase without the need for a contract with the merchant, and they can receive commissions once the transaction is complete and the seven-day no-reason return period has passed. Even if brands do not collaborate with influencers to promote products, influencers can still list and sell brands’ products and earn commissions. The majority of collaborations between influencers and brands are pure commission-based, meaning merchants only pay the influencer commissions for selling their products and do not conduct commercial promotions. Commissions in the selection plaza are transparent, indicating that the initiative for commission income lies with the influencers. The corresponding process for labor cooperation involves brands selecting suitable influencers from “influencer plazas” to discuss business cooperation, where the initiative for this cooperation lies with the brands.

**After revision：**

Labor-service partnerships and commission-based collaborations operate as distinct models. The majority of influencer-brand partnerships function on a pure commission basis: influencers independently select products to sell, earning predetermined commission rates from brands without participating in formal promotional campaigns. In this model, product selection autonomy rests entirely with influencers. In contrast, labor-service partnerships follow a brand-driven process: brands proactively identify preferred influencers to negotiate marketing collaborations. Here, the initiative lies with brands, who dictate partnership terms and campaign objectives.

3.key theoretical foundations like influencer marketing or network game theory are underdeveloped.

**Increased references to the literature on the influencer marketing and network game.**

Add the following literature:

Gurrieri L, Drenten J, Abidin C. Influencer Marketing: Interdisciplinary and Socio-Cultural Perspectives. Taylor & Francis; 2024.

Jiménez-Marín G, Sanz-Marcos P, Tobar-Pesantez L B. Keller’s resonance model in the context of fashion branding: persuasive impact through the figure of the influencer. Academy of Strategic Management Journal. 2021;20: 1–14.

Lee J, Walter N, Hayes JL, Golan GJ. Do Influencers Influence? A Meta-Analytic Comparison of Celebrities and Social Media Influencers Effects. Social Media + Society. 2024;10: 20563051241269269. doi:10.1177/20563051241269269

Sanz Marcos P, Jiménez Marín G, Elías Zambrano R. Aplicación y uso del Modelo de Resonancia o Customer-Based Brand Equity (CBBE). Estudio de la lealtad de marca a través de la figura del influencer. Methaodos revista de ciencias sociales. 2021;9: 200–218.

Zozaya-Durazo, L., Feijoo, B., Sádaba, C. The Role that Influencers Play in Consumption Decisions Made by Spanish Minors. Doxa Comunicación. 2023;36: 401–413.

Perc M, Szolnoki A. Coevolutionary games—A mini review. Biosystems. 2010;99: 109–125. doi:10.1016/j.biosystems.2009.10.003

Santos FC, Pacheco JM, Lenaerts T. Cooperation Prevails When Individuals Adjust Their Social Ties. Amaral L, editor. PLoS Comput Biol. 2006;2: e140. doi:10.1371/journal.pcbi.0020140

Wang Z, Wang L, Szolnoki A, Perc M. Evolutionary games on multilayer networks: a colloquium. Eur Phys J B. 2015;88: 124. doi:10.1140/epjb/e2015-60270-7

4. The reliance on simulations without validation against real-world data limits the practical credibility of the findings.

An experiment with the case of beauty brand sales was added, and the ' Finally, applying reasonable values derived from parameter experiments to the influencer marketing model in the cosmetics industry demonstrates that this approach effectively enhances mutual benefits and stabilizes the overall business environment.' was pointed out in the summary.

**The whole content of the experimental case is as follows：**

**5.3 example verification**

To validate the contribution of our double-layer network model (brand-influencer dynamics) to brand-building, the researchers conducted a case study using the Cosmetics Datasets (https://www.kaggle.com/datasets/kingabzpro/cosmetics-datasets) uploaded by Abid Ali Awan on Kaggle. This empirical analysis demonstrates how the proposed model enhances brand-influencer synergy, providing actionable insights for brand-building strategies.

Due to their broad appeal, high consumption frequency, and accessible price points, beauty products have consistently dominated the online retail sector as a top-selling category. The Cosmetics Datasets document the price points of 1,472 cosmetic products across 101 brands, covering four major product types: moisturizer, cleanser, treatment (targeted skincare), and face mask. Leveraging this dataset, we construct the brand network following these rules:

- **Primary Product Categories & Price Point Calculation:**

For each brand, identify its top two most prevalent product categories (i.e., categories with the highest number of listed products). The average price point (P) of these dominant categories is calculated and assigned as the brand’s representative price.

- **Competitive Relationship Definition:**

An undirected edge is established between two brand nodes if they share at least one primary product category, indicating a competitive relationship within overlapping market segments.

The influencer network is constructed by retaining the top 101 nodes by degree from the post-growth tunable clustering scale-free network. Other model parameters—including node link rules, edge-linking probabilities, and clustering coefficients—are configured using empirically validated values from the aforementioned simulation experiments, as detailed in Tab 5.

**Tab.5 System Simulation Parameter Setting Table**

| Parameter | *x* | *y* | *C1* | *C2* | *Q1* | *P* | *ρ* | *τ* |
| --- | --- | --- | --- | --- | --- | --- | --- | --- |
| initial value | 0.2 | 0.2 | 10 | 1000 | 100 | - | 1 | 0.9 |
| Parameter | *λ* | *α* | *γ* | *R* | *L* | *δ* | *K* | *q* |
| initial value | 5% | 0.1 | 10% | 30% | 400 | log^Ni^ | 0.1 | 0.1 |

In the final experimental phase, profit trajectories for brands in cooperation, brands not in cooperation, influencers in cooperation, and influencers not in cooperation were plotted across the evolutionary timeline, as shown in Fig 12. The horizontal axis represents the game sequence (t), and the vertical axis indicates normalized profit values. The revenue curves of these four agent categories exhibit significant divergence, reflecting distinct strategic outcomes under the proposed model.

Within the brand cohort, brands in cooperation exhibit a robust growth trajectory from the initial stages, indicating a positive correlation between cooperative strategies and economies of scale. In contrast, the profits of brands not in cooperation persistently hover at the zero-profit boundary, demonstrating the invalidation of defection strategy dominance typically observed in the prisoner’s dilemma model.

The influencer cohort exhibits divergent dynamics shaped by strategic cooperation choices. Influencers in cooperation demonstrate profit trajectories characterized by logistic growth, where initial rapid gains reach a local maximum before converging to a suboptimal equilibrium of 1×10⁶ through market-mediated adjustments. This pattern underscores the diminishing marginal returns inherent to cooperative strategies within bilateral markets, as escalating collaboration costs and resource saturation erode incremental benefits. In contrast, influencers not in cooperation sustain profit curves persistently aligned with the zero-value baseline, revealing that creators reliant solely on content production—without brand partnerships—face existential viability challenges in the influencer economy. These contrasting outcomes highlight the critical role of strategic alliances in overcoming market saturation and achieving sustainable monetization, while non-cooperative strategies risk marginalization due to insufficient monetization pathways beyond organic content engagement.

The experimental findings demonstrate that the sustained stability of the influencer economy can indeed generate substantial profits for brands. Notably, influencers’ profits peak earlier than those of brands, aligning with real-world dynamics where the core objective of influencer marketing lies in market expansion and identifying potential loyal customers. Initial profit surges are driven by follower-driven effects, which also explains the subsequent decline in influencer earnings: as the system evolves, the influencer market becomes saturated, and brands acquire a stable base of loyal customers through influencer partnerships. Consequently, both parties’ profits stabilize, reflecting a mature equilibrium in the influencer-brand ecosystem.

**Fig 12 The profit curves of influencers and cosmetics industry brands**

5.As for the writing, there are redundancies and inconsistencies, particularly in the introduction and methodology sections.

The part of the text description of the introduction part is modified to make it simple and remove redundancy.

**Before modified：**

The rapid development of mobile internet has given rise to various content creation platforms, including short videos, forums, live streaming, and information-sharing sites. On these diverse platforms, content creators share their daily lives, perspectives, and ideas through the publication of videos, photos, music, and written content, thereby attracting the attention of internet users. Due to the continuous output of high-quality content and the unique persona tags, these content creators accumulate followers and gradually evolve into influential figures known as “influencers.”

Currently, there is no unified definition for online influencers in the industry; however, they differ from internet celebrities in that they should meet the following four characteristics[1]: (1) Influencers are content creators. They produce content that conveys specific perspectives, thoughts, or emotional value to their audience. (2) Influencers possess a substantial follower base; for instance, Douyin defines creators with over ten thousand followers as influencers. (3) Influencers should have professional knowledge, unique skills, or influence in a specific vertical domain. (4) They must have the ability to assist brands in achieving commercial conversion.

**After revision：**

The proliferation of mobile internet technologies has spurred the growth of content creation platforms—such as short video hubs, live-streaming services, and interactive forums—where creators engage audiences by sharing curated content. Through consistent high-quality output and distinctive personal branding, these creators evolve into influencers, individuals whose expertise and authenticity enable them to wield significant influence within niche domains.

While the term "online influencer" lacks a standardized definition, it is distinguished from "internet celebrity" by four key attributes[1]:(1)Content-Centricity: Production of original material conveying specialized knowledge or emotional resonance.(2)Audience Scale: A follower base meeting platform-specific thresholds.(3)Vertical Expertise: Authority in a specific domain (e.g., fashion, technology).(4)Commercial Efficacy: Demonstrated ability to drive brand promotion.

Special thanks to you for your good comments.

**Responses to the Reviewer’s comments:**

**Reviewer Comments**

**Reviewer #1**

Thank you for the literature provided by the reviewers, which has far-reaching significance for us to further understand and study the economy. We have compiled it into a literature review and written it into the article.

The following is a literature review of the collation：

Influencer marketing, as an emerging promotional paradigm, has garnered significant attention from academia and industry. Gurrieri et al. [16] delve into the complexities of influencer marketing through an interdisciplinary lens, examining how historical, economic, and technological shifts have shaped its evolution. Their work highlights the multifaceted identities of influencers and the strategies non-traditional influencers employ to connect with audiences. Crucially, they argue that influencer marketing has transcended its role as a mere marketing tactic to become a cultural phenomenon, one that both reflects and drives societal trends. Jiménez-Marín et al.[17] focus on the fashion industry, analyzing how influencers shape consumer brand choices. Grounded in Keller’s Resonance Model, their mixed-methods study (combining qualitative and quantitative approaches) demonstrates that influencer-brand collaborations significantly enhance brand equity and consumer loyalty. This research provides empirical validation for integrating influencers into fashion branding strategies. Lee et al. [18] employ meta-analysis to compare the advertising efficacy of social media influencers (SMIs) and traditional celebrity endorsers. Synthesizing 39 experimental studies (2010–2024), they reveal that SMIs achieve comparable or superior results to celebrity endorsers and outperform brand-only campaigns. The study also identifies influencer credibility and tier as critical factors: mega-influencers exhibit stronger persuasive power, while nano-influencers lag in impact—a finding that informs strategic endorser selection. Sanz Marcos et al. [19] further explore how influencers amplify brand loyalty via the Resonance Model. Through in-depth interviews with Instagram influencers and large-scale follower surveys, they show that influencer-brand partnerships strengthen consumer-brand identification and foster long-term loyalty. This work offers empirical evidence for leveraging influencer collaborations to build enduring consumer relationships. Zozaya-Durazo et al. [20] examined influencer marketing strategies targeting minors aged 11–17, a sensitive consumer demographic. Their findings reveal that trust-based relationships between influencers and followers directly shape the perceived credibility of branded content. Notably, younger participants prioritized interactive engagement with influencers, while older adolescents emphasized content quality. These insights underscore the need for stringent ethical guidelines in influencer marketing aimed at minors, urging brands to guard against excessive commercialization that may exert undue influence on youth values.

**Reviewer #2**

Thank the reviewer for your criticism of our work. The depth of our own research questions is indeed insufficient. According to your questions and revision directions, we have made the following modifications to the article.

1. **The paper lack of novel findings.**

**Response to Reviewer Comments:**

We sincerely appreciate the reviewer's insightful feedback. Our study's dual-layer network game model (brand-influencer dynamics) extends beyond conventional game-theoretic frameworks by integrating network topology modulation as a driver of evolutionary game dynamics. Specifically, the parameter tunable clustering (q)—while mathematically representing clustering coefficients—is operationalized within our model to capture real-world influencers' niche specialization and market influence, dimensions that have remained abstract or unquantified in prior literature.

This methodological innovation allows us to dissect how structural properties of influencer networks (e.g., follower concentration patterns) endogenously shape collaborative equilibria—a critical advancement over static game models. We acknowledge that the initial manuscript's abstract did not sufficiently foreground this conceptual contribution. In revision, we will:

Expand the abstract to explicitly articulate the dual-layer model's capacity to bridge network science with behavioral game theory;

Strengthen the discussion section to contrast our q-driven niche specialization metric.

This revision will clarify how our work addresses the gap in quantifying influencer specialization through network topology, a dimension previously overlooked in influencer marketing studies.

**Highlight the role of q in the abstract.**

“Specifically, during the construction of the influencer network, the network structural metric—tunable clustering—is integrated with the practical scenario of uneven follower distribution among influencers, thereby investigating the impact of influencer network clustering intensity on the system’s evolutionary dynamics.”**were added in** **Abstract.**

We found that there was a deviation in the understanding of Tunable clustering ( q ) in the first draft, which led to an error in the experimental results and has now been corrected.Re-verification of the impact of q on the willingness of both parties to cooperate. Revised now. And change the conclusion.

**Before modification：**

**Modified figure：**

**Analysis of the corrected experimental results**

The parameter Tunable Clustering (q) in scale-free networks with adjustable clustering quantifies the degree to which high-degree influencer nodes dominate follower distribution. Specifically, a higher q value leads to a greater proportion of total followers concentrated among top influencers (i.e., nodes with the highest degrees), resulting in a polarized follower distribution where a few "head" influencers attract the majority of followers, while "long-tail" influencers retain minimal audiences. In real-world terms, this implies that influencers with larger follower bases wield greater influence within their vertical niches, as their ability to attract and retain followers reflects both specialized expertise and content appeal. Thus, q indirectly serves as a metric for quantifying an influencer’s niche authority and professionalization level.

As illustrated in Fig 8, structural variations in the influencer network exhibit no discernible impact on brands’ coordination decision-making. However, an increase in the network’s tunable clustering parameter q correlates with a marked decline in influencers’ willingness to partner with brands. This suggests that within the same vertical domain, polarized follower distributions—characterized by a dominance of followers concentrated among a few top influencers—create an environment less conducive to coordination engagements. Such polarization accelerates the attrition of long-tail influencers from the economic ecosystem, destabilizing the equilibrium of the influencer economy by disproportionately favoring "head" influencers. These insights advocate for policy interventions aimed at “de-centralizing” influencer economies, where redistributing economic benefits across all participants, rather than concentrating gains among top influencers, could mitigate systemic imbalances. By fostering inclusivity and equitable resource allocation, such strategies would enhance ecological resilience and drive sustainable macroeconomic growth.

2. **the figures are not well-prepared。**

**Response to Reviewer Comments:**

We admit that the roughness of our drawings in the initial experiment caused a bad reading experience for readers. In the revised version, we redraw all the experimental results, keep the picture size consistent, and make the picture beautiful by extending the coordinate axis range, thickening the lines and legends. The comparison chart before and after the change is as follows :

**Before modification：**

**Modified figure：**

**3.** **improve sections carefully with scientific insights**

**Response to Reviewer Comments:**

**We polished the following language to make the content of the article read scientifically.**

(1) **For the description of the influencer marketing model.**

**Before modified：**

From Fig 1, it can be concluded that the core connection in influencer marketing and the key to monetization lie in the success of collaboration between influencers and brands. The cooperation between the two parties is crucial for forming a closed-loop commercial system. Therefore, studying the cooperative behaviors of influencers and brands is essential for understanding the sustainable development and benefit distribution related to influencer marketing.

**After revision：**

Critically, the sustainability of this ecosystem hinges on effective collaboration between brands and influencers. As illustrated in Fig. 1, their partnership forms the linchpin of a closed-loop system encompassing content creation, followers engagement, and revenue generation. Understanding the dynamics of this cooperation—particularly how micro-level decisions shape macro-level outcomes—is vital for optimizing benefit distribution and ensuring the model’s long-term viability.

**(2) Combined with the previous revisions, the conclusion part is appropriately modified. The main modifications are as follows :**

**Reasons for revision : Integrating the conclusions of the initial manuscript ( 2 ) and ( 3 )**

**Before modified：**

Brands are at a disadvantage in the influencer marketing model. Although the influencer marketing can bring substantial profits to brands, factors related to the products (such as P, C1, C2) have little or no impact on the influencers’ earnings, while relevant variables from influencers greatly affect brands. Brands need to balance their own factors while also assessing indicators related to cooperative influencers to make final cooperation decisions;

**After revision：**

Game model parameters influence cooperative decision-making. Brands face a structural disadvantage within the influencer marketing paradigm: while influencer partnerships can yield substantial profits, product-related factors (e.g., C, C2, γ) exert minimal impact on influencers’ strategic choices yet significantly shape brands’ own decision calculus. Notably, Creator motivation L only affects the decision-making of the master. Therefore, the brand needs to evaluate the relevant indicators with the collaborators while balancing its own factors to make the final cooperation decision.

**Reasons for revision :** The original conclusion ( 3 ) is not strong in the experiment.

**Before modified：**

The results from the study on creative incentives indicate that part-time influencers hold an advantage when the follower count is not high, while full-time influencers are at a disadvantage. Part-time influencers can be considered freelancers to some extent, and their income does not completely rely on brands; full-time influencers, on the other hand, have their income highly linked to business collaborations;

**After revision：**

Network topology significantly influences collaborative decision-making between brands and influencers. The structural parameters of complex networks—such as the tunable clustering coefficient (q), which in this study correlates with an influencer’s niche specialization and market influence—can be abstracted into real-world relational dynamics (e.g., follower concentration, competitive overlaps). These insights underscore the necessity for both brands and influencers to strategically monitor industry landscapes and maintain adaptive agility, recalibrating partnership strategies in response to environmental shifts such as market saturation or algorithm-driven platform changes.
